# Editorial: Sugar reduction strategies in foods: sensory, nutritional and safety evaluation

**DOI:** 10.3389/fnut.2024.1370781

**Published:** 2024-02-07

**Authors:** Dipendra Kumar Mahato, Emmanuela Magriplis, Nitya Sharma, Shirani Gamlath

**Affiliations:** ^1^CASS Food Research Centre, School of Exercise and Nutrition Sciences, Deakin University, Burwood, VIC, Australia; ^2^Department of Food Science and Human Nutrition, Agricultural University of Athens, Athens, Greece; ^3^World Resources Institute India, New Delhi, India

**Keywords:** sugar reduction, sensory, nutrition, health, safety

The surge in health problems attributed to excessive consumption of added sugars encompasses a spectrum of issues, including obesity, diabetes, cardiovascular diseases, and dental cavities. In 2016, statistics from the World Health Organization (WHO) indicated that the worldwide prevalence of obesity was roughly 13%, impacting about 600 million people. This encompassed 1.9 billion adults, comprising 39% of the global adult population, and 340 million children aged five years and older. Projections indicate that if the current growth rate persists, nearly half of the global population could be classified as overweight or obese by 2030. Recognizing the gravity of the situation, the WHO strongly advocates limiting free sugars intake to no more than 10% of total energy consumption, with an even more stringent recommendation of below 5% for dental carry prevention (1). Aligning with these guidelines, Public Health England (PHE) emphasizes the need for a substantial reduction, recommending a 20% decrease in sugar consumption across various food categories.

In the realm of food, sugar assumes a versatile role, serving not only as a sweetening agent but also as a vital component in harmonizing diverse tastes such as sourness, saltiness, and spiciness, especially in less sweet culinary creations. Nevertheless, the reduction of sugar presents potential challenges that may affect customer acceptance, alter physicochemical qualities, and impact food safety (2). The intricate involvement of sugar in gastronomy extends beyond mere enhancement of taste. Its presence holds significance in achieving the desired texture, bestowing an appealing color, infusing distinct flavor notes, and contributing to the preservation of food items. As endeavors are undertaken to decrease sugar content in food and beverages, it becomes imperative to carefully consider the ramifications on these essential aspects.

Reducing sugar content without compromising the sensory attributes of food requires careful consideration of appropriate reduction techniques and the incorporation of suitable sugar alternatives (3). Striking a balance between achieving health-conscious sugar reductions and maintaining the sensory and safety characteristics of food is crucial for successful implementation of sugar reduction strategies in the food industry. Consequently, a thoughtful and strategic approach is necessary to navigate the intricate web of factors involved in sugar reduction, ensuring that the end products not only meet health standards but also retain their sensory appeal and safety.

In essence, these recommendations underscore the imperative to address the public health challenges associated with excessive sugar intake (4). By advocating for significant reductions in sugar content across a range of commonly consumed foods, health organizations aim to mitigate the rising prevalence of conditions linked to high sugar consumption, find ways to reduce this intake and assess the healthfulness of these, and promote overall wellbeing. This Research Topic presents comprehensive and remarkable reviews and original articles on sensory, nutritional and safety and health aspects of sugar reduction and sweeteners trends.

Authors from around the globe have submitted their manuscripts for consideration in this Research Topic (Alharthi et al.; Griebel-Thompson et al.; Cramer et al.; Pappe et al.; Grilo et al.; Yeung et al.; Chen et al.). Alharthi et al. uncovered that among healthy participants, there was a heightened awareness regarding the use and potential drawbacks of artificial sweeteners. Moreover, through bivariate analysis employing logistic regression, significant correlations were found with variables such as gender, age, and educational attainment. The study underscores the importance of educational initiatives to promote safe consumption of artificial sweeteners, particularly among females, emphasizing the need for tailored nutritional guidance and awareness campaigns to mitigate potential health risks associated with their usage, a pattern that might be prevalent not only in Saudi Arabia but also in other regions globally.

Griebel-Thompson et al. highlighted that while previous studies have identified circumstantial factors (such as sociodemographic influences) or indirect mechanisms (like maternal lifestyle and modeling) contributing to elevated added sugar intakes in infants and toddlers, their study introduces the notion of exposure to added sugars from infant formula as a potential direct mechanism. This sheds light on potential reasons certain infants and toddlers may consume higher amounts of added sugars, suggesting a need for further investigation into the formulation and regulation of infant formulas to mitigate potential health risks associated with excessive sugar consumption in early childhood. Cramer et al., on the other hand, propose clinical trials with adequate duration and pertinent clinical endpoints to explore the significance of erythritol (along with other sugar substitutes) in the context of cardiovascular disease development. This is essential for obtaining clearer insights into their potential impacts on health and guiding future dietary recommendations and interventions. Pappe et al. reported that reducing the intake of free sugars in the diet leads to a decrease in body weight and body fat, potentially linked to a reduction in total energy intake. However, this dietary change does not influence the daily mean glucose levels and glycaemic variability in individuals without diabetes. Additional research exploring its effects on overall metabolic health parameters, beyond weight management, could offer a more comprehensive understanding of the impacts of sugar reduction on health outcomes.

Grilo et al. emphasize the urgency of addressing the proliferation of non-nutritive sweetener (NNS) in children's beverages, advocating for enhanced transparency in labeling and rigorous oversight to safeguard children's health. Furthermore, the study encourages stakeholders, including policymakers, healthcare professionals, and parents, to collaborate in promoting informed dietary choices and advocating for clearer guidelines that prioritize children's wellbeing. Grilo's research serves as a catalyst for ongoing dialogue and action to ensure that children have access to wholesome beverages that contribute positively to their overall health and development. Similarly, Yeung et al. provide a comprehensive examination of scholarly research trends surrounding monk fruit extract and mogrosides. The analysis reveals a growing interest in these natural sweeteners within the scientific community, reflecting a shift toward healthier alternatives to traditional sugar. As consumer demand for low-calorie and natural sweetening options continues to rise, the findings underscore the importance of ongoing research and development in exploring the potential benefits and applications of monk fruit extract and mogrosides in various industries.

Further, Chen et al.'s suggestion to optimize NNS use, especially for individuals with diabetes, highlights the importance of tailored educational interventions targeting those with limited knowledge, attitude, and practice, signaling the necessity for further research to explore the effectiveness and long-term implications of such interventions in promoting healthier dietary choices among diabetic individuals. In conclusion, the exploration of sugar, types of sugar, and sugar substitutes remains ongoing across diverse populations, yielding valuable insights that can significantly advance relevant research and investigations in this field.

The Guest Editor team would like to extend heartfelt gratitude for the valuable contributions to this Research Topic. We are delighted with the intriguing research and findings centered around sweeteners and health aspects. Furthermore, we are grateful for the invitation and the opportunity to edit this Research Topic, made possible through the tremendous support of the editorial team at Frontiers in Nutrition.

## Author contributions

DKM: Conceptualization, Writing—original draft, Writing—review & editing. EM: Writing—review & editing. NS: Writing—review & editing. SG: Conceptualization, Writing—review & editing.
